# Interventions for Prevention of Intimate Partner Violence Against Women in Humanitarian Settings: A Protocol for a Systematic Review

**DOI:** 10.1371/currents.dis.f41d45fbdca13babe4ae5be0f9732e75

**Published:** 2017-07-12

**Authors:** Marjan Delkhosh, Ali Ardalan, Abbas Rahimiforoushani, Abbas Keshtkar, Leila Amiri Farahani, Effat Merghati Khoei

**Affiliations:** Department of Disaster Public Health, School of Public Health, Tehran University of Medical Sciences, Tehran, Iran; Department of Disaster & Emergency Health, National Institute of Health Research, Tehran University of Medical Sciences, Tehran, Iran; Department of Disaster & Emergency Health, National Institute of Health Research, Tehran University of Medical Sciences, Tehran, Iran; Department of Disaster Public Health, School of Public Health, Tehran University of Medical Sciences, Tehran, Iran; Harvard Humanitarian Initiative, Harvard University, Cambridge, MA, USA; Department of Health Sciences Education Development, School of Public Health, Tehran University of Medical Sciences, Tehran, IRANTehran University of Medical Sciences; Department of Midwifery, School of Nursing and Midwifery, Iran University of Medical Sciences, Tehran, Iran

## Abstract

**Introduction:**

Humanitarian emergencies and the number of people who are adversely affected are increasing. In such emergencies, the vulnerability of women and girls to gender-based violence increases signifi­cantly and they often experience high levels of intimate partner violence (IPV). There are a limited number of interventions to reduce gender-based violence (GBV) and IPV in the contexts of humanitarian emergencies, and there is uncertainty about the effectiveness of these preventive interventions. This is the protocol for a systematic review that will synthesize the evidence on interventions for primary or secondary prevention of IPV in humanitarian settings, and assess the effect of existing types of IPV-related interventions in these settings.

**Methods and Design:**

The PRISMA-P 2015 statement has been used to prepare this report. Studies published from January 2000 to January 2017 will be reviewed with no language limits. Any experimental, quasi-experimental, or controlled trials will be included. A combination of four key concepts, including “IPV” AND “population” AND “humanitarian setting” AND “intervention” will be used in the search and a variety of information sources will be used: (1) bibliographic databases; (2) special databases and grey literature; (3) and the reference lists of eligible studies. Two reviewers will independently screen articles, extract relevant data and assess study quality. Discrepancies will be resolved through consensus. Risk of bias will be assessed using the Cochrane Risk of Bias tool and the quality of evidence will be assessed using the CONSORT checklist. A narrative synthesis will be provided. If a sufficient number of studies are found, their results will be pooled using a random-effects meta-analysis. For dichotomous outcomes, summaries of intervention effects for each study will be provided by calculating risk ratios with 95% confidence interval. Standardized mean differences will be used for continuous outcomes.

**Discussion:**

The review will be useful for IPV management policy and related planning. It will help researchers, policymakers and guideline developers with an interest in reducing violence against women among refugees, internally displaced persons (IDPs), and conflict-affected population.

## Background

Almost, 11 million people were registered as refugees or internally displaced persons (IDPs) across the world in January 2012, in an estimate from UNHCR 1. The refugee population reached an unprecedented 19·6 million individuals worldwide in 2015 and the number is steadily increasing 2^,^^3^. UNHCR also reported the highest number of forcibly displaced people in recorded history in 2015: approximately 59.5 million 4. Humanitarian emergencies and the number of people adversely affected are increasing 5. In September 2016, at a Summit in the United Nations General Assembly, the “New York Declaration for Refugees and Migrants” was adopted expressing the political will of world leaders to save the lives of refugees and migrants, protect their rights, and share responsibility for large movements on a global scale. One of the main commitments of this document was the prevention and response to sexual and gender-based violence 6. Violence against women in conflict-affected settings and complex emergencies is a serious global public health issue 7^,^^8^. During periods of conflict or displacement, the vulnerability of women and girls to sexual and gender-based violence increases signifi­cantly 9. Research has shown that approximately one in five refugees or displaced women experienced sexual violence alone 1. Also, emerging prevalence data show that intimate partner violence (IPV) is relatively common in these settings 10. IPV is the most prevalent form of gender-based violence 11 and is an important global public health problem 12.Women are the most common population suffering from IPV and adverse impacts are widely reported among women who experienced IPV 13^,^^14^^,^^15^^,^^16^^,^^17^^,^^18^ ^19^. The Global Burden of Disease Study and WHO (2013) estimates that over 30% of women age 15 or over have experienced some kinds of IPV in their life, such as physical or sexual IPV 8^,^^20^^,^^21^. Conflict-affected, refugee and internally displaced women often experience high levels of IPV 7. According to some studies, the prevalence of IPV among conflict-affected communities in Bosnia and Herzegovina, Serbia, East Timor, and the Democratic Republic of Congo (DRC) ranged from 24 to 76% 22. In other studies, IPV prevalence among Palestinian refugees and conflict affected displaced women on the Thai–Burma were between 22 to 42% and nearly 8%, respectively 22.

The increases in the number of humanitarian emergencies and growing number of refugee, IDPs and affected people related to them, makes research in this field are an international policy concern and a research evidence priority 5.

Despite the increasing number of humanitarian emergencies 5, increasing IPV occurrence in these settings 21, and IPV related adverse health outcomes 19^,^^22^^,^^23^^,^^24^^,^^25^^,^^26^, current knowledge on how to prevent IPV is limited 10. Some of the interventions or strategies that have been implemented are women empowerment, gender equity, group training, advocacy and psychosocial support interventions 27.

There are a limited number of evaluations of interventions aimed at reducing GBV and IPV in the contexts of humanitarian emergencies and refugee contexts 27^,^^28^^,^^29^. Although there has been an increase in the number of programs in refugee populations to prevent and respond to GBV, particularly sexual violence against women and girls, there remains a general lack of evidence on the effectiveness of these efforts (prevention programs, interventions, and strategies, especially among refugee populations) in preventing diverse forms of GBV, and a lack of evaluation of efforts outside of conflict-related sexual violence 28. There is a need for accessible research that evaluates the efficacy and effectiveness of various GBV prevention and management strategies in refugee and displaced populations 29.

This is the protocol for a systematic review that will synthesize information on interventions for primary or secondary prevention of IPV against women in humanitarian settings. It will also investigate the effect of existing types of interventions on prevention or reduction of physical, sexual or emotional IPV in these settings.

## Objectives of study

The objective of the systematic review is to identify existing IPV prevention interventions, strategies and programs among refugee, internally displaced, or conflict-affected female in humanitarian settings; and to assess their effects. It will seek to answer the following questions:

1. What are the existing types of intervention for primary or secondary prevention of IPV against women in humanitarian settings?
2. What is the effect of various types of interventions on prevention or reduction of physical, sexual or emotional IPV in humanitarian settings?
3. How do various types of intervention related to IPV affect health outcomes in humanitarian settings?

## Methods

The Preferred Reporting Items for Systematic reviews and Meta-Analyses for Protocols 2015 (PRISMA-P 2015) statement has been used for preparing and reporting of this systematic review protocol 30.

******Registration******

This systematic review was registered in the International Prospective Register of Systematic Reviews (PROSPERO) on 18 September 2016 and updated in the registry on 15 December 2016 (CRD42016047497 available at http://www.crd.york.ac.uk/PROSPERO).

**Eligibility criteria**

Studies will be selected according to the criteria outlined below.

**Study designs**

Any experimental, quasi-experimental, or controlled trials studies (randomized trials or quasi-randomised trials) will be included.

**Participants**

The population will be restricted to refugee, internally displaced, or conflict-affected women aged 15 years and over who live in the situation of humanitarian settings. According to UNHCR, IDPs are defined as “persons or groups of persons who have been forced or obliged to flee or to leave their homes or places of habitual residence, in particular as a result of, or in order to avoid, the effects of armed conflict, situations of generalized violence, violations of human rights or natural or human-made disasters, and who have not crossed an internationally recognized state border” 31.Also, refugees are defined as people who have been forced to flee their country because of persecution, war, armed conflict or violence 32. In this review, refugees and IDPs will be defined as people who have been displaced or fled within or outside their home countries because of armed conflict and other situations of violence (not related to natural disasters). We define “conflict-affected persons” as persons residing in active or recent conflict zones or in a post-conflict settings 33^,^^34^. People Affected by Conflict (PAC) will be considered, including those who are forcibly or not forcibly displaced and Conflict Affected Residence (CARs) who, for any reason, did not flee or leave their homes or places of habitual residence 31.

Studies in which it is not clear if the study population were refugees or migrants will be excluded. Studies that only focused on displaced population associated with natural disasters will be excluded. There will be no limits on study participants in terms of country or ethnicity. All refugees or IDPs who relocated in high-income country settings will be eligible for this review.

**Interventions**

IPV will include physical, sexual, or emotional forms of violence, abuse or battering between intimate partners. We define IPV-related interventions as any type of intervention conducted in a humanitarian setting for women or men in order to prevent or reduce violence against a woman partner or its related health outcomes. The studies need to have a clear pre/post intervention evaluative component in order to be able to assess its effectiveness. Also, the interventions should focus on efforts that targeted the primary or secondary prevention of IPV or its related health outcomes, including efforts to prevent initial victimization or re-victimization in defined population and settings. Primary prevention aims to prevent initial IPV and secondary prevention aims to prevent or reduce ongoing IPV or its related health outcomes 35.

Eligible interventions include individual interventions; population-, group- or community-based prevention interventions; single or multi-component interventions and programs and may be delivered at various levels 36^,^^37^.Intervention types may include, but not be limited to: educational programs, group training, survivor care, advocacy, psychosocial support, home visitation, batterer intervention, livelihood interventions or strategies, community mobilization, personnel interventions, cash transfers, police advocacy, legal strategies, systems and security interventions, and other forms of IPV-related intervention 27^,^^35^^,^^37^^,^^38^.

Interventions that aim to address and modify health-related outcomes following IPV will also be included.

**Comparators**

Any comparison group (such as no intervention, or an alternative intervention) will be eligible.

**Types of outcome measures**

In this systematic review, the primary outcome related to IPV-intervention will be prevention or reduction in male-to-female physical, sexual or emotional IPV or changes in severity, frequency or types of physical, sexual or emotional IPV against affected women after the intervention in humanitarian settings (e.g. reduced sexual abuse).

Changes in health outcomes which occur following the implementation of the interventions will be regarded as secondary outcomes for assessing the impact of intervention. These include, but are not limited to:

- changes in physical health outcomes related to IPV, including decreased physical health sequela (such as deaths [decreased mortality related to IPV]), decreased injuries and changes in acute or chronic health disorders [decreased morbidity related to IPV] 13^,^^14^^,^^15 ^
- changes in psychological/mental health outcomes related to IPV (such as improvement in mental well-being, reduced depressive symptoms) 16 17;
- changes in sexual and reproductive health (SRH) outcomes related to IPV (including decreased incident of HIV/STIs and reproductive disorders, unwanted or mistimed pregnancy; pregnancy coercion, pregnancy termination; improvement in contraceptive use, improvement in sexual health behaviors)^18^^,^^19^.

Some other changes following the intervention will also be regarded as secondary outcomes. These include:

reduction in the occurrence of any other type of IPV (e.g. economical violence), changes in behaviors (e.g. improvement in controlling behaviors, improvement in healthcare use, use of IPV and community-based resources/referrals, improvement in health/help seeking behaviors), changes in attitudes or social norms (such as reduced acceptability of spousal violence among men, equitable gender norms), increased financial autonomy and security among women, improved economic well-being, enhanced relationship quality, empowerment of women, and promotion of health and health-related quality of life 21^,^^24^^,^^27^^,^^35^^,^^37^.

The debate about appropriate outcomes for IPV intervention studies, means that we will not use outcomes measures to determine study eligibility 35.

**Settings**

A humanitarian setting is one in which an event or series of events has resulted in a critical threat to the health, safety, security or well-being of a community or other large group of people. This can be the result of events such as armed conflicts, natural disasters, epidemics or famine, and often involves population displacement 39. In this review, the situation of humanitarian settings is defined using the criteria specified by the Sphere Standards as a range of situations including conflict and complex political emergencies in all countries (including low- or middle-income countries) 40^,^^41^. Eligible studies will be those conducted in humanitarian settings, that is contexts affected by war, terrorist attack, political violence or armed conflict.

Refugee/IDP camps or settlements will be eligible settings. All refugees, IDPs, or conflict-affected people who are living in non-camp settings (e.g urban, pre-urban, or rural area) will be included.

**Data and Language:**

No language limits will be imposed on the search. Studies published between January 2000 and January 2017 will be sought.

**Exclusion criteria:**

Studies not primarily about the prevention or reduction of IPV or its health-related outcomes will be excluded from this review; as will studies that do not describe an eligible population or intervention. The exclusion criteria include (1) not about IPV (other GBV topics e.g., female genital mutilation, forced or early marriage, rape); (2) female-to-male IPV; (3) not about refugees, IDPs, or conflict-affected; (4) not describing prevention interventions or strategies; (5) not describing or measuring the outcomes of the intervention; (6) no baseline data for comparison; (7) qualitative studies and descriptive quantitative papers with no specific intervention and no outcomes; (8) letters, editorials, commentaries, reviews; and (9) tertiary prevention.

**Information sources**

A variety of information sources will be used to identify studies meeting the inclusion criteria: (1) electronic bibliographic databases for published studies, using a comprehensive search; (2) special list databases and grey literature; and (3) the reference lists of studies included in the review.

**Electronic bibliographic databases**

Published literature in the following databases will be searched: PubMed (MEDLINE), Web of sciences, Scopus, Cochrane Library ((The Cochrane Central Register of Controlled Trials (CENTRAL)), PsycINFO, ProQuest, and google scholar.

The dates of the search will be recorded for each database at the time the search is done.

**Gray literature**

The following databases and websites will be searched for reports and unpublished papers: WHO Global Health Library, UNHCR database, Reproductive Health Response in Crisis Consortium (RHRC), Inter-Agency Working Group on Reproductive Health in Crisis (IAWG), and the Reproductive Health Access Information and Services in Emergencies (RAISE). The PROSPERO registry of systematic reviews will be searched for registered reviews in this field. To ensure literature saturation, reference lists of included studies will be checked.

Documentation of the information sources, including the name of each source, the date range searched (start and end dates) and the search platform (for electronic database searches) will be recorded.

**Key search terms and Search strategy**

Medical Subject Headings (MeSH), key terms and text-based search terms will be adapted from prior published literature. A combination of four key concepts (main key terms and their synonyms) will be used: “IPV” AND “population” AND “humanitarian setting” AND “intervention”.

The main search terms will be used in different combinations, using the Boolean operators (“AND” and “OR”) and wildcard variants depending on the database being searched. In searching, the Boolean operator “OR” will be used to link each key concept to their synonyms. Then, all key concepts will be combined using “AND” to identify relevant literature. The searches with these defined key words will be limited to the title, keywords or abstract of the article. Search terms will be combined in the following manner:

(All ‘IPV’ terms connected by the search term OR) AND (all ‘population’ terms connected by the search term OR) AND (all ‘humanitarian setting’ terms connected by the search term OR) AND (all ‘intervention’ terms connected by the search term OR). Table shows the key search terms. The full search strategy used for two main databases will be presented in the final report. The initial proposal for the search strategy for PubMed is in Appendix A. This strategy will be applied to the other electronic databases where relevant, modified as necessary.

**Figure:**
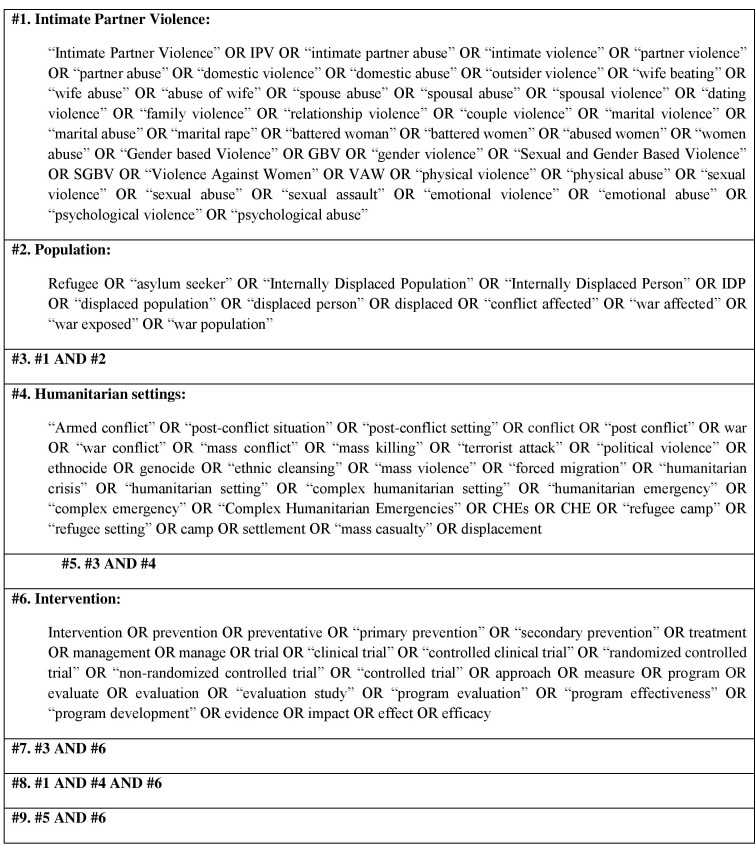
Table 1: Key search terms that will be used and their combination.

**Data management**

All search results will be downloaded and saved into reference management software (EndNote version X7).

**Study selection**

The description of study selection in final report will be according to the PRISMA guidelines, including the PRISMA flow diagram 42.

Two reviewers (MD and LA) will read the titles and abstracts of citations retrieved from searching, after exclusion of duplicate records, to remove those that clearly do not meet the inclusion criteria or are outside the scope of the review. The abstract of retrieved papers that are in a language other than English will be translated using Google Translate and then will be judged. The same two reviewers will read the full text of potentially relevant studies and confirm their eligibility. In the event of any uncertainty, a third reviewer (EM) will be consulted. The decision of a third reviewer will determine study eligibility. An electronic file that lists excluded references by primary reason for exclusion will be maintained.

**Data extraction**

Data will be extracted from included papers by using a standardized pre-piloted data extraction form. This form will be adapted from Cochrane Public Health Group’s “Guide for Developing a Cochrane Protocol” 43. The data extraction form will be piloted by two reviewers (MD and LA) on a set of the studies to assess the feasibility of the use of this tool, check its adequacy and consider if any revisions or changes are needed. All included studies will be reviewed and coded according to their relevance. Data extraction will be conducted for all included articles by one team member (MD) and verified by a second (LA). Disagreements between reviewers will be resolved by discussion.

**Data items**

Data will be extracted for the following:

Study ID/characteristics (first author name, year of publication, evaluated country), participants/population type (e.g. refugee, IDPs), socio-demographics characteristics of participants (e.g. age, Gender, Place, Race, ethnicity, Religion, Education), study setting/situation (e.g. refugee camp, settlement, urban/pre-urban refugee settings, conflict-affected settings, etc.), specific criteria for inclusion or exclusion, study design (e.g. experimental or quasi-experimental), sample sizes, intervention type and description (intervention details), total number of intervention groups, frequency and duration of interventions (exposure period), the specific goals of the interventions, the duration of follow-up, intervention impact, retention rates, control group types and descriptions, outcome definitions and measurement tools, main findings/results, miscellaneous, and key conclusions including implications for practice and/or research from each study. Potential discrepancies will be resolved by consensus.

**Risk of bias/quality assessment**

Two review authors (AK and MD) will independently assess the possible risk of bias for each included studies by using the Cochrane Collaboration’s Risk of Bias (RoB) tool according to the following domains:

- Randomization sequence generation

- Treatment allocation concealment

- Blinding

- Completeness of outcome data

- Selective outcome reporting

- Other sources of bias 44

Each included study will be rated as low risk, high risk, or unclear and the rating of each study will be included in an appendix in the final report. If insufficient detail is reported for a study, the risk of bias will judged as ‘unclear’. Any disagreements between the reviewers over the risk of bias will be resolved by discussion and consulting with a third author (AA).

A quality appraisal of the full texts of eligible papers will be done using the CONSORT checklist to assess methodological quality 45. A pilot trial on two included studies will be conducted to assess the feasibility of using this tool in this review. Study quality will be independently appraised by two reviewers (MD and LA). Papers will be ranked as high, medium or low quality rating based on the checklist criteria. If any disagreements arise, a third reviewer will be consulted (AA).

**Data synthesis and analysis:**

A narrative synthesis of the findings from the included studies will be provided. Key findings related to each type of prevention intervention will be analyzed separately and results will then be combined to provide an overall synthesis of publications related to interventions to prevent IPV in humanitarian settings. The narrative synthesis of the findings will focus on target population characteristics, characteristics of included studies (for example, type of humanitarian emergency and study setting), type and content of interventions, duration of the intervention, effectiveness of interventions, types of outcomes measured and findings.

Quantitative synthesis will be used if the included studies are sufficiently homogeneous and comparable across interventions and outcomes measured. Whether or not a meta-analysis is possible, a summary of findings table will be used to present the findings for each primary outcome.

However, it is anticipated that there will be limited scope for meta-analysis because of the range of different outcomes measured across the small number of existing trials 46. If a sufficient number of articles with the same type of intervention and outcome measure are found, the results of these studies will be pooled using a random effects meta-analysis. For dichotomous outcomes, we will provide summaries of intervention effects for each study by calculating risk ratios (RR) with 95% confidence interval (CI). We will use standardized mean differences (with 95% CI) for continuous outcomes.

**Dealing with missing data**

When there are missing data, we will attempt to contact the original authors of the study (by using email addresses on the study’s publication) to obtain the relevant missing data.

**Analysis of subgroups or subsets**

If sufficient data are available, subgroup analyses will be used to explore possible sources of heterogeneity, based on different types of interventions.

**Reporting of results**

This protocol followed the PRISMA-P Statement 47 . and the final report of the systematic review will be presented according to the PRISMA guidelines. For ensuring transparency, a PRISMA flow chart, 48 a table of included studies and the reasons for exclusion of studies will be clearly documented as Figure 1.

**PRISMA Flow Diagram:**
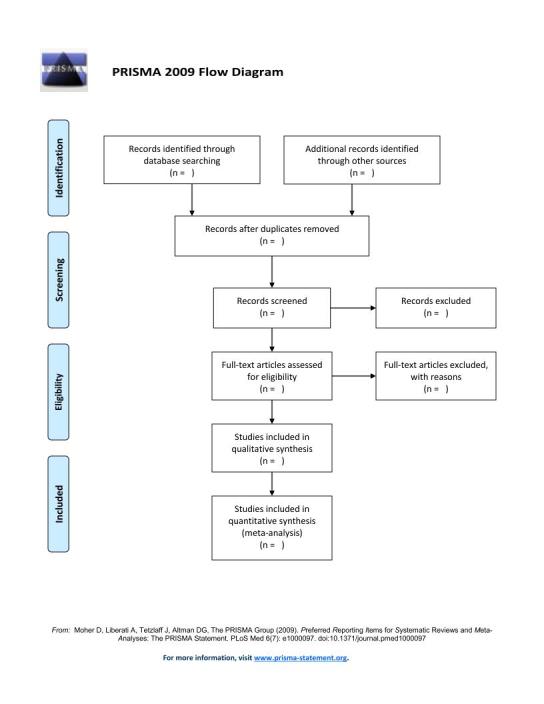

**Interpretation of findings**

The results of the review will be discussed in the context of the quality of evidence, and the limitations and the strengths of findings, with emphasis on general interpretation of the results in the context of other evidence and their implications for IPV management practices (including IPV-related intervention, strategies and programs) and for future research in humanitarian settings.

## Discussion

The number of people affected by humanitarian emergencies is increasing and making it an international policy concern and a research evidence priority 5. Despite the increasing number of refugee and displaced population following humanitarian emergencies 49, and the high level of different types of GBV in these settings 50, ncluding IPV, there is a lack of evidence about IPV-related intervention and their effectiveness in humanitarian settings. Also, notwithstanding growing attention to IPV globally, systematic evaluation of evidence for IPV prevention remains limited 35, particularly after humanitarian emergencies. Therefore, the limited and scattered evidence in the literature supports the need for a systematic review in this regard 51. This review will provide a detailed summary of the existing evidence on the implementation and effectiveness of various used IPV-related interventions to prevent or reduce occurring or reoccurring IPV against refugee, displaced and conflict-affected women. The evidence will be useful for IPV management policy and related planning. The review will also help researchers, policymakers and guideline developers with an interest in reducing violence against women associated with the humanitarian setting.

One of the strengths of this review is the use of a systematic approach and employing reliable and robust tools such as PRISMA-P 2015 47. Although systematic reviews of randomized trials are considered to be the highest level of evidence for assessing the effects of interventions 52, restricting our review in this way would be limited by methodological challenges 52^,^^53^. Such trials are difficult to implement in the setting of humanitarian emergencies and we may not be able to find sufficient trials to provide users of this review with a body of useful information 53. To overcome this challenge, we will search a wide range of databases and sources, after piloting and revising the search strategy 54.

**Ethics**

As this protocol is for a systematic review, formal ethics committee review is not required.

**Review output and dissemination plans**

Academic review products will be produced, critically appraising the implementation and effectiveness of interventions targeting prevention or reduction of IPV against women affected by humanitarian emergencies. The systematic review articles will be submitted to academic journals for peer-reviewed publication and the findings will be presented at conferences and seminars.

## Authors’ contributions:

MD is the guarantor and conceived the original research idea with guidance from EM. MD developed the first draft of the protocol. AA, EM and AK oversaw the development and revision of the first draft of the protocol and contributed to revisions. MD, LA and AK contributed to the development of the selection criteria, and the strategy for the assessment of risk of bias assessment and data extraction. MD, AK and LA will develop the search strategy. MD and LA will extract data from studies. MD and AK will assess the risk of bias for each included study. AA and AK will provide statistical expertise, perform and interpret the analysis. EM and AK will resolve any disagreements. MD will draft the final report of the systematic review. All authors read and approved this manuscript, and will do the same for the final report of the review.

## Data Availability Statement

All relevant data are within the manuscript and the public repository Figshare: http://figshare.com/s/cb2feda97952c89eec8e. For more information, please contact the author Marjan Delkhosh (delkhoshmarjan@gmail.com).

## Competing Interest Statement

The authors have declared that no competing interests exist.

## Corresponding author

Effat Merghati Khoei, PhD. Iranian National Center of Addiction Studies (INCAS), Iranian Institute for Reduction of High-Risk Behaviors, Tehran University of Medical Sciences, Tehran, Iran.

Brian & Spinal Cord Injury, Neuroscience Institution, Tehran University of Medical Sciences, Tehran, Iran. Email: effat_mer@yahoo.com

## Appendix

**Figure:**
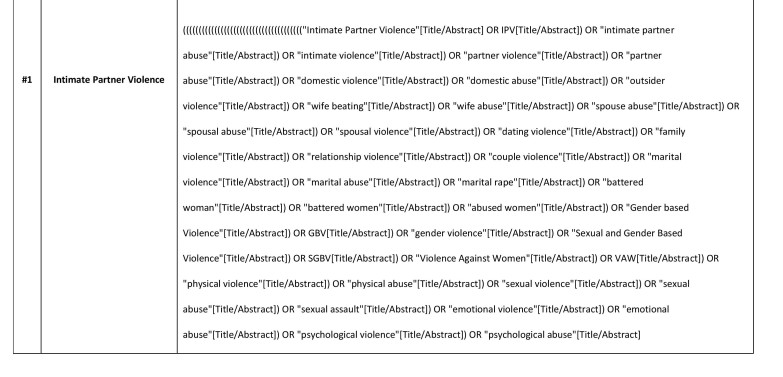
Appendix A: Sample search strategy in PubMed

**Figure:**
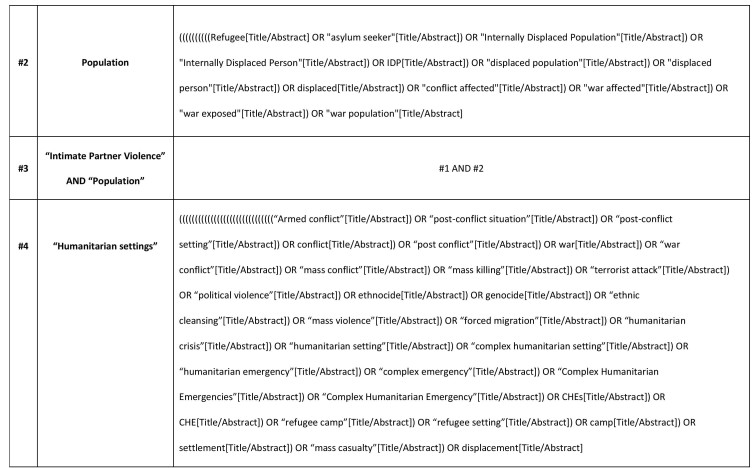
Appendix A: Sample search strategy in PubMed

**Figure:**
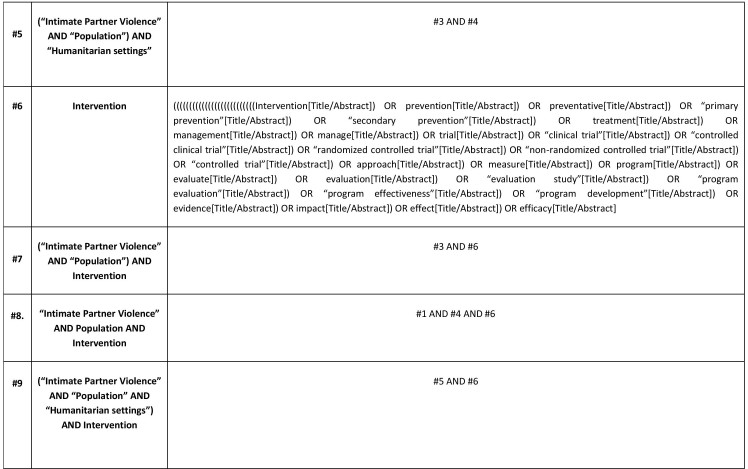
Appendix A: Sample search strategy in PubMed
